# Improved early-stage crop classification using a novel fusion-based machine learning approach with Sentinel-2A and Landsat 8–9 data

**DOI:** 10.1007/s10661-025-14420-9

**Published:** 2025-08-06

**Authors:** Muhammad Daniyal Jamil, Muhammad Zahid Abbas, Muhammad Farhan Saeed, Aftab Jamal, Muhammad Mubeen, Ali Zakir, Iftikhar Ahmad, Rimsha Jameel, Katarzyna Pentoś, Yaser Hassan Dewir, Jakub Černý

**Affiliations:** 1 Department of Computer Science, COMSATS University Islamabad, Vehari Campus, Vehari, 61100 Pakistan; 2 Department of Environmental Sciences, COMSATS University Islamabad, Vehari Campus, Vehari, 61100 Pakistan; 3 Department of Soil and Environmental Sciences, Faculty of Crop Production Sciences, The University of Agriculture, Peshawar, 25130 Pakistan; 4 Institute of Agricultural Engineering, Wroclaw University of Environmental and Life Sciences, 37B Chełmonskiego Street, 51-630 Wrocław, Poland; 5 Department of Plant Production, College of Food and Agriculture Sciences, King Saud University, 11451 Riyadh, Saudi Arabia; 6 Department of Silviculture, Faculty of Forestry and Wood Technology, Mendel University, Zemědělská 3, 613 00 Brno, Czech Republic; 7 Department of Biotechnology, COMSATS University Islamabad, Vehari Campus, 61100 Vehari, Pakistan

**Keywords:** Image fusion, Feature integration, Multi-patch GLCM, Crop classification, Deep learning, Early-stage crop

## Abstract

Crop classification during the early stages is challenging because of the striking similarity in spectral and texture features among various crops. To improve classification accuracy, this study proposes a novel fusion-based deep learning approach. The approach integrates textural and spectral features from a fused dataset generated by merging Landsat 8–9 and Sentinel-2A data using the Gram-Schmidt fusion approach. The textural features were extracted using the multi-patch Gray Level Co-occurrence Matrix (GLCM) technique. The spectral features, namely the Enhanced Vegetation Index (EVI) and Normalized Difference Vegetation Index (NDVI), were obtained using the spectral index method. The five machine learning methods (deep neural network, 1D convolutional neural network, decision tree, support vector machine, and random forest) were trained using textural and spectral parameters to develop classifiers. The proposed approach achieves promising results using deep neural network (DNN), with an accuracy of 0.89, precision of 0.88, recall of 0.91, and F1-score of 0.90. These results demonstrate the effectiveness of the fusion-based deep learning approach in enhancing classification accuracy for early-stage crops.

## Introduction

Recently, remote sensing technology has gained popularity in agriculture. It offers cost-effective and timely data for monitoring crops, predicting yields, and classifying crops (Demelash et al., 2021). Remote sensing technology offers different platforms, making it possible to detect and monitor spatial and temporal activities of any area whose data is required accordingly. The most commonly used remote platforms in monitoring land use and land cover (LULC) are satellites (e.g., Sentinel-1, Sentinel-2, Landsat 8, and MODIS).

Numerous studies have been conducted to classify crops on remotely sensed satellite images using deep learning (DL) and machine learning (ML) methods, achieving incredible results. Satellite image results differ based on their spatial and temporal resolutions. As an image’s spatial resolution grows, its pixel area shrinks, revealing more of the image’s characteristics in each pixel. It identifies the smallest pixel area that can be determined (Mulla, 2013). The temporal resolution provides a time series method, which makes it easy to classify crops from time to time at different phenological stages (Inglada et al., 2016; Messina et al., 2020; Yi et al., 2020). In recent years, crop classification has become more accessible due to increased high-resolution and multispectral images. The Landsat-8 satellite, having a spatial resolution of 30 m and a total revisit duration of 15–16 days, is also used in crop classification. Landsat-8 provides multispectral (MS) images that help to classify and differentiate the data of the required crop from other crops in the same area. Recently, the Sentinel-2A satellite also provides multi-spectral imagery data, which is available publicly for monitoring land use and land cover (Wang et al., 2022). It has multiple sensors mounted on it, which cover 13 spectral bands ranging from visible to near-infrared (NIR) and short-wave infrared (SWIR) wavelengths, with a spatial resolution of 10 m and a total coverage duration of 5–10 days (Messina et al., 2020).

Numerous studies have been conducted to understand how Sentinel and Landsat time series data contributed to agriculture. To examine the effectiveness of multiple ML classifiers in crop-type mapping using different image set combinations for pre-harvest crop classification using Sentinel-2 data. As in a study by Li et al. (2022), U-Net has been applied for crop type mapping in early growth periods in the Hetao irrigation district in China using time-series Sentinel-2 imagery. A study by Maponya et al. (2020) reveals that pre-harvest photos can identify crops accurately, with solid classification accuracies (77.2%) obtained 8 weeks before harvest using support vector machine (SVM) and random forest (RF) classifiers. Sentinel-2’s potential was explored for identifying early crops in Northeast China in another study. The findings of the experiments showed that B12, NDTI, LSWI, and NDSVI were critical in the early stages of crop identification (Wei et al., 2022a, 2022b). To accurately classify the crops at different stages of their growth, various studies introduced deep-learning techniques and the use of satellite time series data. Using Sentinel-2 time series data, Wang et al. (2021) integrate crop geography information into a better Convolutional Block Attention Module (CBAM), called Geo-CBAM. To enhance the CNN model’s spectral and spatial attention, the Geo-CBAM module is included. The model naturally assigned varied weights to the qualities, with red-edge values in the middle of the year receiving greater attention. In another study, Seydi et al. (2022) proposed an attention module, a deep learning CNN model that makes use of a dual-attention convolutional neural network, which has a Dam for the classification of crops using time series Sentinel-2 imagery. The proposed DAM includes both spectral and spatial attention modules, improving feature extraction based on crop patterns and spatial arrangements. A high level of accuracy was obtained with the help of the proposed framework and the performance surpassed such models as RF, XGBoost, R-CNN, and CBAM, which points to the efficiency of the approach, which can be used in remote sensing for accurate crop-type differentiation. Whereas to categorize winter wheat in Shandong, China, Zhang et al. (2021) provided an automated early-season method (AEMMS) based on Sentinel-2 time-series data. The AEMMS technique predicts that the winter wheat in the area is in the early season phenological phase and has the largest biomass increase of all the winter crops. Some studies explore the potential of DL neural networks, such as the row anchor selection classification technique (RASCM; Wei et al., 2022a, 2022b) for early-stage crop row-following utilizing GhostNet, a lightweight deep convolutional neural network (DCNN). In another study, the transformer framework crop classification has been carried out for Sentinel-1 and Sentinel-2 data. This model does not include temporal interpolation in intra-season classification; it obtains high F1 scores (greater than 0.8 for pre-harvest classification) and may be applied to ends like yield estimation and water balance modeling (Weilandt et al., 2023). In another study, three DL models for early-stage crop categorization using Sentinel-1A SAR were used: 1-Dimensional Convolutional Neural Networks (1DCNNs), Long Short-Term Memory (LSTM) Recurrent Neural Networks (RNNs), and Gated Recurrent Unit (GRU)-RNNs (Zhao et al., 2019a, 2019b). The findings demonstrate that 1D CNNs attained a Kappa coefficient of 94.2%, while the GRU RNNs accomplished the maximum significant value of 0.934 more quickly than other classifiers. In one study, Conv1D and Sentinel-2 data with a 5-day interval achieved an overall accuracy of 0.83 for early-season crop mapping in the Shiyang River Basin (Yi et al., 2022). Using Sentinel-2A data, automated approaches were introduced to map crop types. In a study by Nasrallah et al. (2018) to map winter wheat using high-resolution Sentinel-2 imagery, a unique approach was introduced: Simple and Effective Wheat Mapping Approach (SEWMA). SEWMA is a tree-like technique that uses the Normalized Difference Vegetation Index (NDVI) readings over four months corresponding to various phenological stages of wheat to detect crop growth that uses cadastral data and satellite images (Hsiou et al., 2022).

Apart from using Sentinel data, different studies have been conducted using Landsat satellite imagery data because of its high temporal resolution, which provides detailed information on the phenological cycles of crops. In one study, an EVI-based decision tree mapping approach for mapping paddy rice fields was presented. The study looked at how the quantity and temporal distribution of Landsat pictures affected classification precision. Multi-temporal photos can differentiate between early, late, and double-cropping rice only and medium rice with an overall accuracy range between 71.8% and 85.8% (Cao et al., 2021). In another study performed by Zhong et al. (2019), two DL models were created, one based on Conv1D layers and the other on LSTM. The outcomes demonstrated that the Conv1D-based model outperformed LSTM and non-deep-learning classifiers, achieving an F1 score of 0.73 and maximum accuracy of 85.5% using Landsat Enhanced Vegetation Index (EVI) time series. In another study, an end-to-end architecture that uses an LSTM-RNN model to enhance and streamline land cover mapping was used to achieve an overall accuracy of 97.0% (Sun et al., 2019). Multi-temporal data using deep learning approaches have also shown the potential for crop classification (Xia et al., 2022; Zhang et al., 2018). The integration of the Landsat time series and the Common Land Unit (CLU) was conducted to solve the cloud interference issue. It was observed during the study that combining Landsat image data and CLU enhanced classification accuracy (Cai et al., 2018). The integration of Landsat 7/8 and Sentinel-2A/B time series photos suggests a new technique for mapping cropping intensity. The method extracts the entire growth cycle and considers crop phenology. This method is helpful for accurately capturing the changes in the planting patterns of cropland (Pan et al., 2021). The use of satellite image time series (SITS) in combination with high spatial and temporal resolution optical data was employed for early crop-type detection. Combining Landsat 8 SITS with Sentinel-1 SAR photo time series (Inglada et al., 2016), the authors discovered that classification accuracy might improve significantly, enabling earlier land cover mapping than when optical images are used alone. In another study, a digital elevation model (DEM) for mapping irrigated crops using Sentinel-1 SAR image series and Landsat 8 optical images was introduced. The findings suggest that combining multiple data sources (radar, optical, and DEM) enhance early classifications of irrigated crops, with a kappa coefficient of 0.89 compared to 0.84 for sorts using each data type independently. However, as crops reach maturity, the DEM is no longer relevant (Demarez et al., 2019). However, another study by Kordi and Yousefi (2022) combined time series optical, SAR, and digital elevation model (DEM) data. The findings of that study demonstrated that using ideal Landsat 8 spectral bands with vegetation indices and Sentinel-1 polarization channels with extra textural information gave excellent classification accuracy of 77.0% and high accuracy of 89.0% for the two datasets, respectively.

Several studies considering the importance of features for crop classification showed the importance of features. The most commonly evaluated features are spectral and textural, impacting the crop’s discriminative characteristics. Various studies showed that short-wave infrared (SWIR-1) and red-edge band 1 (RE-1) spectral features produced superior classification proficiency (Yi et al., 2020; Zhang et al., 2020). However, the selection of suitable features is important to achieve acceptable results. To select the best features, different studies suggest different feature selection models, such as Phenology-based Spectral and Temporal Feature Selection (PSTFS) proposed by Hu et al. (2019). The PSTFS approach used MODIS data from Heilongjiang Province in China in 2011 to create an excellent map of maize that had an 85% accuracy rate. Seydi et al. (2021) proposed a method to assess wildfire impacts over Australia using Sentinel-2 and MODIS images in Google Earth Engine (GEE). It used change detection and machine learning approaches to generate binary maps of fire scars and mapped a new feature selection technique with an average accuracy of 91.0%. Although the overall effectiveness of the approach was proven for large-scale burned area mapping, the use of the MODIS LULC product had a relatively low resolution for the land cover classification. Seydi et al. (2024) further enhanced the evaluation of SBS by developing a physics-constrained machine learning model that combines VBS with climatology, meteorology, and ecology. It scored an accuracy of 89.0% and underscores how the differenced normalized burn ratio (dNBR) and climatic factors affecting SBS are essential to guide post-fire management and recovery efforts. In another study, a hybrid CNN-RF network was introduced. The optimum spectral bands for crop mapping were chosen using the optimal feature selection method (OFSM), which was shown to perform better overall accuracy than the two conventional feature selection techniques, such as RF feature integration and RF-random feature elimination (RF-FI and RF-RFE; Yang et al., 2020). The random forest classifier with spectral indices produces more precise results (Rahmati et al., 2022). In the study of Kobayashi et al. (2020), classification accuracy was significantly increased, reaching a total accuracy of 93.1%. The findings of the study suggested that a total of eight spectral indices resulted in achieving such accuracy. However, some studies (e.g., Fei et al., 2022; Tang et al., 2018) showed that NIR displays the most value among spectral features for the classification of crops. However, using an individual type of feature limits, various spectral and textural data were utilized indicating that exploring different features showed the importance of texture features alongside spectral features (Fei et al., 2022; Wang et al., 2022).

The Gray Level Co-occurrence Matrix (GLCM)–based texture features play a very important role in distinguishing the crops at their initial stages. In one study, the accuracy was improved by more than 10.0% when textural information was added to the SAR data (Koley and Chockalingam, 2022). Whereas in another research, spectral and textural elements derived from SAR and optical data for calculating maize Leaf Area Index (LAI) and biomass were integrated. The findings of the experiment showed that maize LAI and biomass estimates were much more accurate thanks to texture features, while SAR texture features greatly impacted biomass estimation (Luo et al., 2020). While using the Gray Level Run Length Matrix (GLRM) and Local Binary Pattern (LBP) algorithms to retrieve textural features, classify various Iranian wheat kinds with around 95.0% accuracy. The study showed that approaches for extracting texture information from images and processing that information accordingly could result in achieving greater accuracy (Khojastehnazhand and Roostaei, 2022). Several studies were conducted using color and texture for crop classification (Mekhalfa and Yacef, 2021; Muneer and Fati, 2020; Yuan et al., 2019).

Identifying crop types at their early growth stages is difficult because of their texture and color similarity (Iqbal et al., 2021). Classifying crops in their early phases is challenging because there are not many characteristics that can be used to differentiate between the many types of crops at these early stages of growth. Various agricultural applications, such as determining cultivated lands, projecting yields, and conducting prompt interventions for effective resource allocation and management, depend on accurate classification at the initial stages.

Therefore, this study is focused on a novel fusion-based approach for early-stage crop classification. The approach captures a thorough representation of crop properties, enabling improved identification between two different crop varieties (wheat and maize) by incorporating both textural and spectral signals from a fused dataset. The Feature Weighting Method (FWM) is also used to improve classification accuracy by enhancing the derived features’ capacity to discriminate between different classes. The dataset produced by the merger of Landsat 8–9 and Sentinel-2A data offers improved spatial and temporal resolution, enabling the detection of minute alterations in crop properties throughout their early growth phases. The main contribution of this research is developing a fusion-based strategy using a deep-learning approach for early-stage crop classification. To achieve that, the feature extraction from fused data was performed using multi-patch GLCM and spectral indices method. After this, the integration was done to get the best features for the classifier to learn and get accurate results.

## Materials and methods

### Study area

**Fig. 1:**
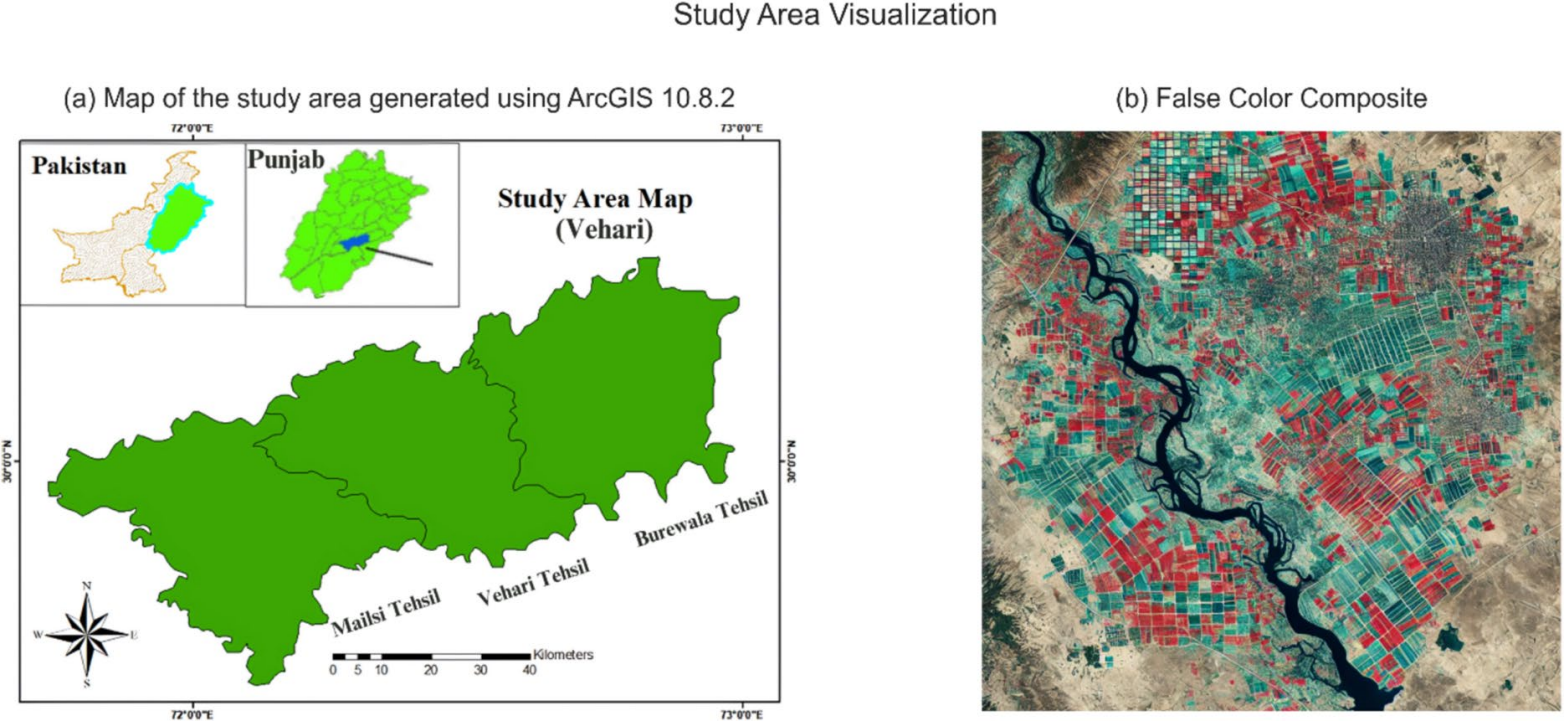
Map of the study area generated using ArcGIS 10.8.2 (**a**) and false-color composite image of the region (**b**)

The study area selected for this research is the District Vehari of Southern Punjab (Pakistan). This area lies between latitude 29°3,505,100 N to 30°2,202,100 N and longitude 71°4,305,400 E to 72°5,804,300 E approximately (Fig. 1a). District Vehari is geographically characterized by fertile land and semi-arid climatic conditions. The District Vehari agricultural landscape offers a distinctive environment for researching early-stage crop classification. The area is well suited for studying the process of crop classification and monitoring throughout the early phases of growth since it undergoes different seasonal variations and crop growth patterns. The months of June, July, and August exhibit the highest air temperatures, with mean values ranging from 38 to 48 °C. The region is irrigated by the waters of the rivers Ravi and Sutlej, and its primary crops include rice, wheat, maize, sugarcane, and cotton (Saeed et al., 2020).

### Dataset

#### Landsat time-series data

Satellite imagery from Landsat 8–9 that was gathered as part of the L1 Collection makes up the dataset used in this study. Landsat Path 15 and Row 39 cover the study area. To provide temporal coverage and capture the dynamic changes in the research area’s vegetation over time, the Landsat imagery is captured at regular intervals. The Landsat provides a temporal resolution at 8-day intervals. This temporal data is essential for early crop classification because it makes it possible to recognize various growth phases and phenological patterns in crops. The dataset of a total span of December 2021 to April 2022, consisting of 317 Landsat images, was created. A thorough investigation of the development of crops during the early stages can be performed using the dataset. The Landsat imagery under the L1 Collection offers a high spatial resolution of 30 m and captures data in several spectral bands, including the visible, near-infrared, and shortwave infrared regions of the electromagnetic spectrum. Table 1 shows the details of the seven bands that are used in this study. The dataset was collected from the site (https://earthexplorer.usgs.gov/), which is available publicly.

**Table 1:** Detailed information of seven bands from Landsat 8–9

Band number	Description	Wavelength	Resolution
Band 2	Visible blue	0.450 to 0.515 µm	30 m
Band 3	Visible green	0.525 to 0.600 µm	30 m
Band 4	Visible red	0.630 to 0.680 µm	30 m
Band 5	Near-infrared	0.845 to 0.885 µm	30 m
Band 6	Short wavelength infrared	1.56 to 1.66 µm	30 m
Band 7	Short wavelength infrared	2.10 to 2.30 µm	60 m
Band 8	Panchromatic	0.50 to 0.68 µm	15 m

#### Sentinel time-series data

The Sentinel-2A satellite is also used to get data for the study area and provides images at a high spatial resolution of 10 m with a temporal resolution of 5 days and 13 spectral bands. The level-2A surface reflectance is used to get atmospherically corrected images of the study area. A total of 348 images were collected over the regular period of the Sentinel-2A temporal resolution. Temporal dynamics make the dataset more suitable for early-stage crop classification. The data was collected based on the crop calendar of the study area from December 2021 to April 2022. This multi-spectral information provides valuable insights into crop health, vigor, and composition. Table 2 shows the details of spectral bands used for the dataset. The data of Sentinel-2A is collected from the site (https://dataspace.copernicus.eu/browser/), which is publicly available.

**Table 2:** Sentinel-2A spectral bands

Band	Resolution	Central Wavelength	Description
B2	10 m	490 nm	Blue
B3	10 m	560 nm	Green
B4	10 m	665 nm	Red
B5	20 m	705 nm	Visible and near infrared (VNIR)
B8	10 m	842 nm	VNIR
B8a	20 m	865 nm	VNIR
B11	20 m	1610 nm	Short-wave infrared (SWIR)
B12	20 m	2190 nm	SWIR

#### Ground truth data

Ground truth information was gathered from 16 separate field stations inside the study region of District Vehari, Pakistan. Gathering ground truth data acts as a standard for calibrating and confirming the outcomes of the remote sensing study. Field surveys were conducted to gather ground truth information. To achieve reliable geo-referencing of the ground truth data, exact position coordinates were also captured using mobile GPS applications.

### Data preparation

**Fig. 2:**
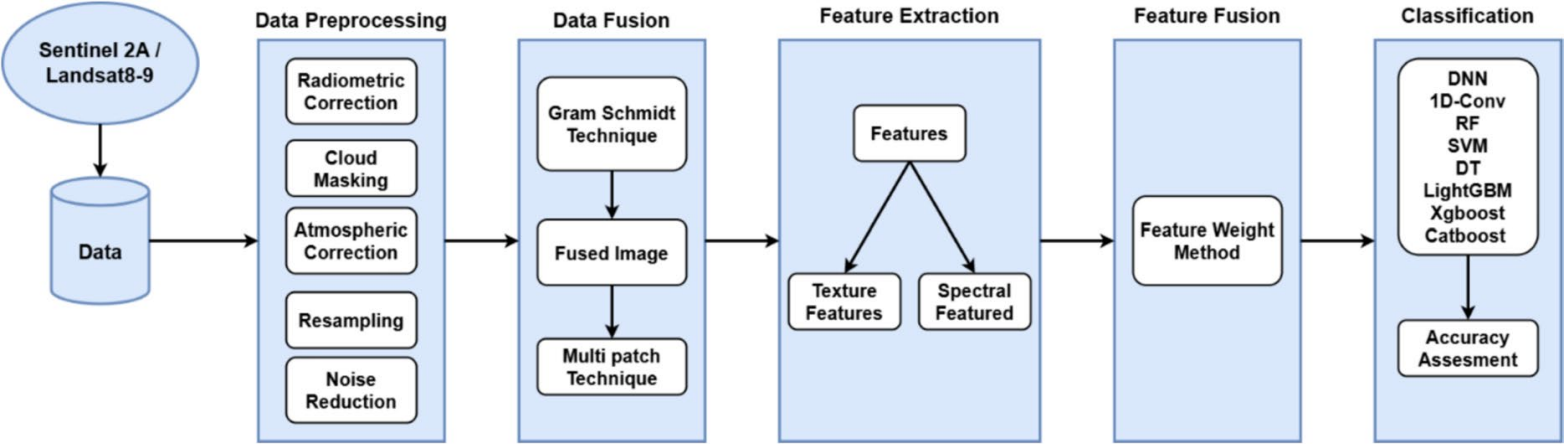
Architectural flow chart of proposed methodology

The proposed solution mainly focused on the classification problem of crops at early stages in complex environments. The methodology is divided into the following steps: (1) data preprocessing, (2) data fusion, (3) feature extraction, (4) feature fusion, and (5) classification, which can be seen in Fig. 2.

#### Data preprocessing

**Fig. 3:**
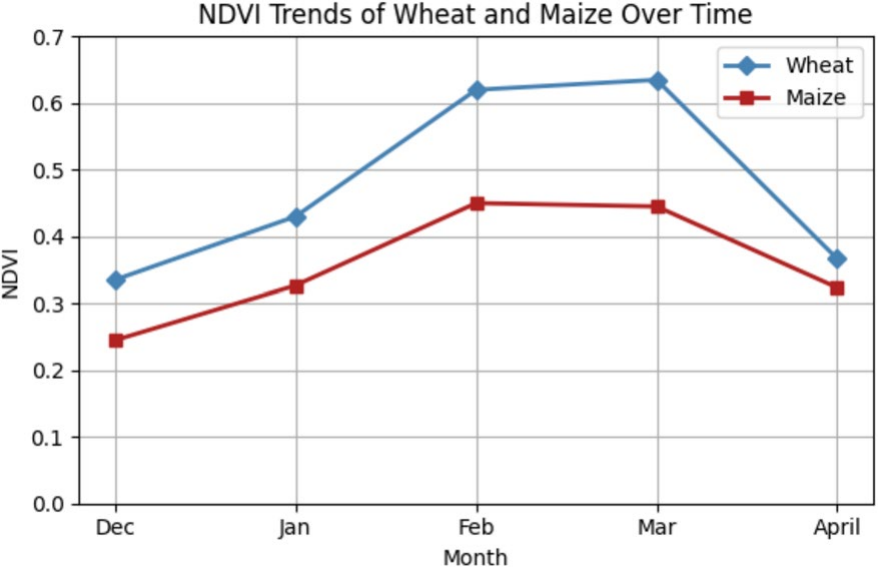
The graph illustrates the monthly average change in NDVI for wheat and maize over five months. Wheat grows most actively in March, reaching its highest level. Maize values for NDVI are even lower, which indicates differences in the crop’s time of growth

The framework of the suggested fusion-based deep learning solution for early-stage crop classification includes preprocessing, which is a crucial step. It is designed to improve the quality of the incoming data and get it ready for additional analysis and feature extraction. The noise or irregularities in the input data were eliminated using denoising and outlier detection methods. For the improved presentation of vegetative cover and types of land use, an image with NIR, red, and green bands was raster created into a false-color combination. Figure 1b consists of the composite image in which red pixels represent healthy vegetation conditions while the other colors represent other land characteristics. Radiometric calibration by Yang et al., (2020) was done using ArcGIS (10.8.2) to uniformly scale the pixel values across various sensors or images. This stage takes the changing weather, illumination, and sensitivity into account for the before and after effects. The atmospheric correction was conducted using dark object subtraction, which is the simplest and most popular method for adjusting the atmospherics of an image. This technique counts on the presence of a surface reflectance of zero or very little. All pixels are deducted from the minimum DN value found in a scene’s histogram. The next step of data preprocessing was image resampling. Sentinel-2A and Landsat 8–9 images have different spatial resolutions of 10 m and 30 m, respectively. To ensure spatial consistency and uniform pixel size, cubic convolution resampling was done. A weighted average of the 16 pixels that are closest to the input coordinates is employed in the cubic convolution technique to identify the grey levels of an image. After that, the output coordinates are given the resulting value. In general, people prefer this approach to bilinear interpolation. Sentinel-2A and Landsat 8–9 spectral bands were used to produce new composite images that emphasize particular features crucial for the classification process (Fig. 3).

#### Data fusion

Data fusion was done after preprocessing using Gram-Schmidt fusion techniques Zhao et al., (2019a, 2019b). This fusion approach makes sure that the redundant and correlated information from the input images gets discarded, while the fused image retains the spectrum information from the input images. The technique produces a fused image by orthogonalizing the input images and captures the distinctive spectral properties of each input image, making it appropriate for purposes such as early-stage crop categorization. It is fast and easy to deploy and provides highly integrated images. This technique produces the panchromatic band for Sentinel-2A and the low synthetic resolution panchromatic band from Landsat images. It does that by taking the mean values of all the bands of images. In this fusion technique, the first principal component is removed, and the information is distributed among all pixels. It is particularly important for improving the spatial and spectral properties of the original datasets as applied in the Gram-Schmidt fusion technique. The fused image from Landsat’s panchromatic band and Sentinel-2A’s spectral bands maintains both high spatial and spectral resolution, which were not achievable in the standalone images of the two satellites. It has to be noted that the orthogonalization process maintains nominal spectral distortion, so the merged dataset is suitable for highly accurate tasks, for example, crop classification.

In particular, the method retains items like vegetation indices and texture, which are crucial for the precise identification of various crops. This enables us to have an accurate fused image that represents the heterogeneity of agricultural fields. Also, the process helps to reduce noise and duplicity and integrates the fused dataset in that it is computationally tractable for analysis.

#### Feature extraction

This step performs the extraction of texture and spectral features from the fused dataset. The texture feature extraction was conducted using GLCM, and spectral features were generated against Vegetation Indices (VI). The multi-patch GLCM technique is an image patch-based technique by Moazzam et al. (2021) used for texture feature extraction. This technique captures pixel-by-pixel information of the whole image. The sliding window approach is used, and a window size of 15 pixels inclusive of four patch statistics is used to capture the patch-by-patch information of the image. This technique moves systematically across the image, aimed at extracting textural as well as spatial information in small areas referred to as patches. Therefore, all patches give local information that enhances classification accuracy, particularly in zones that contain growth boundaries (Haryanto et al., 2021; Wu et al., 2022). Hence, GLCM was applied to extract the texture features. The most commonly extracted features using GLCM are correlation (COR), contrast (CON), energy (angular second moment ASM), and entropy (ENT). GLCM provides pixel information based on angle orientation and the distance of the pixels from each other. The robust characterization of texture required extracting features correlation, contrast, energy, and entropy from GLCM, whereas four different angular orientations (0°, 45°, 90°, and 135°) were used for this purpose.

In this study, the scenario was designed to cover different angles and assign adequate distance so that good and reliable texture features were extracted. The mean measured values from texture features along these angular directions appear in Table 3.

**Table 3:** Mean and standard deviation of GLCM features at different orientations

GLCM feature	0° (Mean ± SD)	45° (Mean ± SD)	90° (Mean ± SD)	135° (Mean ± SD)
Correlation	0.31 ± 0.02	0.28 ± 0.01	0.32 ± 0.03	0.30 ± 0.02
Contrast	0.25 ± 0.02	0.23 ± 0.01	0.26 ± 0.02	0.24 ± 0.02
Energy	0.15 ± 0.02	0.14 ± 0.01	0.16 ± 0.01	0.15 ± 0.01
Entropy	0.29 ± 0.03	0.27 ± 0.02	0.30 ± 0.03	0.28 ± 0.02

The texture variables are expressed in Table 4.

**Table 4:** Spectral and spatial indices

Index name	Equation			Description
NDVI	$NDVI=\frac{NIR-Red}{NIR+Red}$		(1)	This index is an arithmetic balance of NIR and red reflectance; refers to Normalized Difference Vegetation Index. Higher values suggest a healthy amount of vegetation
EVI	$EVI=2.5\bullet \frac{NIR-Red}{NIR+6\bullet Red-7.5\bullet Blue+1}$		(2)	Improved NDVI; superior to the enhanced vegetation index as it negates the impact of aerosols, or atmospheric, canopy influence. G, *C*1, C2, L for correction
`Correlation	$Correlation={\sum }_{i=1}^{N}{\sum }_{j=1}^{N}\frac{\left(i-\mu \right)\left(j-\nu \right)P(i,j)}{{\sigma }_{i}{\sigma }_{j}}$		(3)	Calculates the correlation of pixel pairs; larger correlation values are usually seen in uniform texture which can be used to determine the structure of crops
Contrast	$Contrast={\sum }_{i=1}^{N}{\sum }_{j=1}^{N}{\left(i-j\right)}^{2}P(i,j)$		(4)	Conveys the difference between the pixel value of a location and that of its surrounding pixels; large values signify high contrast and convey regions of interest within crop fields
Energy	$Energy={\sum }_{i=1}^{N}{\sum }_{j=1}^{N}{P(i,j)}^{2}$		(5)	Also termed as angular second moment; this parameter outlines how the textural variation occurs across the particular region of interest, with its larger value implying reduced variance in the texture common in any given area of uniformly growing crops
Entropy	$Entropy=-{\sum }_{i=1}^{N}{\sum }_{j=1}^{N}P\left(i,j\right)log(P\left(i,j\right))$		(6)	Calculates pixel intensity dispersion; higher entropies imply variation in texture for crops to distinguish them

**Fig. 4:**
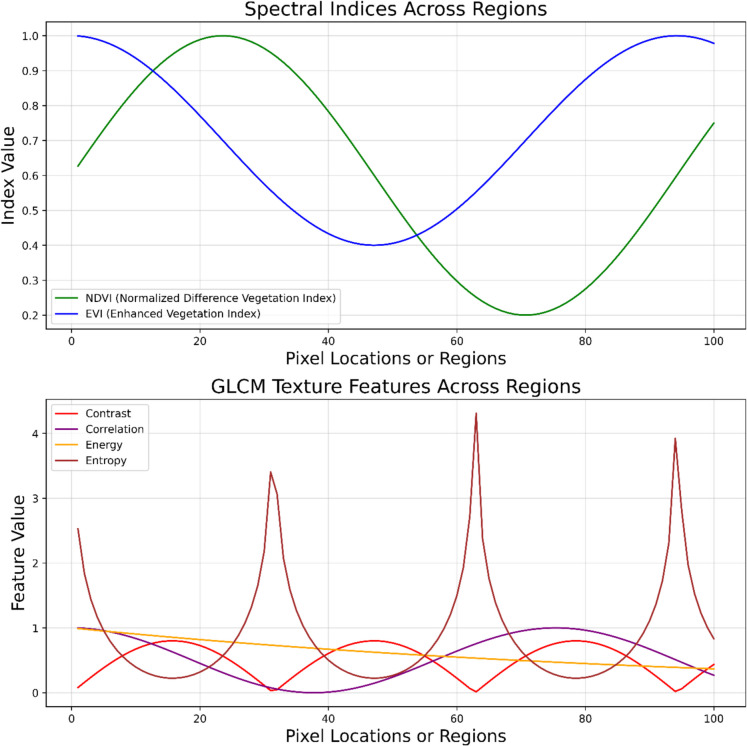
Spectral and texture feature variation

In Eqs. 1–4, (*i*,*j*)th represents the component of normalized symmetric of the co-occurrence matrix. *μ* and *ν* values are constants that correspond to the “mean” or “center” of the distribution. They show how distant each index is from the center values when subtracted from the relevant indices,* i* and *j*. The variables connected to indices *i* and *j* are represented by a joint probability *P(i,j)*. This signifies the covariance between the two variables in the context of correlation, whereas *N* represents the maximum summation possible over which *i* and *j* are summed (Fig. 4).

The spectral index method is used to extract spectral features. Spectral indices are mathematical algorithms that combine distinct bands from multispectral photographs to highlight unique traits of vegetation or crops. These indicators can offer useful details regarding the density, water content, and other crucial characteristics of the vegetation. The Enhanced Vegetation Index (EVI) and Normalized Difference Vegetation Index (NDVI) are used in this study. EVI was selected because of its ability to describe changes in canopy structure and vegetation conditions at the beginning and middle periods of crop development. EVI lessens the influence of the atmosphere and the canopy, while the difference makes EVI applicable in crop identification in complex terrains (Hao et al., 2020). The mathematical representations for both EVI and NDVI are mentioned in Eqs. 5 and 6, respectively. All equations of spectral and spatial indices with some technical descriptions are given in Table 4.

The six features described above (correlation, contrast, energy, entropy, EVI, and NDVI) were used as input parameters for classifiers employed in this research. The five datasets, according to various time series, were constructed: t1 (40,000 vectors obtained after data fusion and feature extraction carried out on images from December 11 to January 25), t2 (45,000 vectors obtained based on images from January 29 to February 15), t3 (50,000 vectors obtained based on images from February 19 to March 10), t4 (55,000 vectors obtained based on images from March 14 to April 6), and t5 (60,000 vectors obtained based on images from April 10 to April 30). These vectors are developed as the outcome of data fusion and feature extraction from the satellite images acquired at different times. Each vector is for the set of attributes derived from the related images, which enable a fine-grained temporal analysis of crop phases. From patches of the fused image, vectors were extracted, and each patch represented a particular time-series interval. This was done from ground truth determination of the various crop types to ensure each vector contained the right information about the crop type at a given development stage. This leads to time series datasets with vectors encompassing spectral as well as textual data that help achieve accurate classification at various growth stages of crops. All classification tools, described in the “Classification methods” section, were trained using T1–T5 datasets. For this process, datasets were randomly divided into training and validation sets in proportion of 80:20, detail by time interval with training, validation, and test set sizes are presented in Table 5.

**Table 5:** Detail of datasets by time interval with training, validation, and test set size

Time interval	Period covered	Total samples	Training set size (80%)	Validation set size (10%)	Test set size (10%)
T1	Dec 11–Jan 25	40,000	32,000	4000	4000
T2	Jan 29–Feb 15	45,000	36,000	4500	4500
T3	Feb 19–Mar 10	50,000	40,000	5000	5000
T4	Mar 14–Apr 6	55,000	44,000	5500	5500
T5	Apr 10–Apr 30	60,000	48,000	6000	6000

#### Feature fusion

The Feature Weighting Method (FWM) was used to validate the weighting parameters for spectral and textural information. The FWM calculates the standardized distance automatically and modifies the weights between spectral and textural parameters following the standardized distance expressed in Eq. 7. The following sections describe the specific steps involved in using FWM to combine spectral and textural features:

Creating a sequence of connections between the GLCM textural variables and the VI variables, where the rows represent the variables and the columns depict how each variable’s values have changed over time.

Determining each variable’s mean and variance values as well as the standardized distance between each pair of variables.

Using the sum of the spectral and texture features in proportion, calculate the weighting factors and the standardized distance between each pair of features.

7 $${d}_{\text{norm}}=\frac{{x}_{i}-{x}_{j}}{{w}_{i}+{w}_{j}}$$

where* d*_norm_ represents the standardized distance, *x*_*i*_ and *x*_*j*_ are the mean values, and *w*_*i*_ and *w*_*j*_ are the standard deviations of each kind of variable, respectively.

Assuming that the feature expressions of all variables are *X*_original_ = [*x*_1_*n, x*_1_2 *….x*_1_*m, x*_2_1*, x*_2_2*, x*_2_*n]*, and *m* and *n* are the quantities of the spectral and textural variables, respectively. If the weighting factors for spectral information and textural variables are represented as ${w}_{1}$ and ${w}_{2}$, then the following formulas can be obtained:

8 $${w}_{1}=\frac{{\sum }_{k=1}^{m}{d}_{k}/m\bullet (m+n)}{{\sum }_{k=1}^{m+n}{d}_{k}}$$

9 $${w}_{2}=\frac{{\sum }_{k=n+1}^{m+n}{d}_{k}/m\bullet (m+n)}{{\sum }_{k=1}^{m+n}{d}_{k}}$$

where *m* and *n* are the numbers of spectral indices, and *d*_*k*_ is the standardized distance between each variable calculated from Eq. 7.

In this research, the novelty is in using image fusion and feature fusion concurrently in a one-stop manner, which is relatively unique within the first phase of crop classification. In previous work, image fusion and feature fusion have been employed separately, whereas we create a compact method for improving both spatial as well as spectral resolution by integrating the two stages in our approach. This integration permits the fused dataset to maintain the high spatial resolution that is lost when employing the per-band PCA while maintaining the spectral features, which is beneficial for achieving a high level of accuracy for characterization and particularly for the early crop stages, where it is difficult to get an accurate classification. This additional fusion step enabled us to set up an adequate feature vector that encompasses not only spectral but also spatial properties of crops.

After the fusion stage, the resulting feature vector is of size six dimensions, which involves two vegetation indices (EVI and NDVI) and four textural features of correlation, contrast, energy, and entropy images from both Sentinel-2A and Landsat 8–9 data. These dimensions describe important aspects of the spectral and textural appearance of crops at the early stages of their development. Because of the higher order of correspondence and dependency of these features, a deep neural network (DNN) is used for classification. As for the fused features, the DNN’s multilevel architecture enables it to acquire complex relationships between them. This proves that DNN is vital in dealing with the fused high-dimension feature space in the early crop classification stage.

The quantitative analysis of the fusion-based methodology involved comparing features obtained from individual data sources and combined features through the system, as presented in Table 6.

**Table 6:** Comparison of original and fused features

Feature name	Original dataset (Mean ± SD)	Fused dataset (Mean ± SD)	Improvement (%)
NDVI	0.62 ± 0.07	0.78 ± 0.04	+ 25.8%
EVI	0.45 ± 0.05	0.61 ± 0.03	+ 35.5%
Correlation	0.43 ± 0.04	0.63 ± 0.03	+ 46.5%
Contrast	0.15 ± 0.02	0.27 ± 0.01	+ 80.0%
Energy	0.11 ± 0.01	0.18 ± 0.02	+ 63.6%
Entropy	0.41 ± 0.05	0.27 ± 0.02	− 34.1%

A quantitative evaluation of spectral and textural features obtained from analysis confirmed that Gram-Schmidt fusion yielded exceptional results. The spectral indices exhibited substantial positive changes under the Gram-Schmidt fusion method—NDVI rose 25.8% from 0.62 ± 0.07 to 0.78 ± 0.04, while EVI improved 35.5% from 0.45 ± 0.05 to 0.61 ± 0.03. These results indicated a heightened precision of vegetation mapping because the method minimized atmospheric distortion and enhanced canopy visual readability. The textural feature analysis based on multiple-patch GLCM revealed meaningful improvements since it led to a 46.5% increase in correlation values, which demonstrates more coherent patterns across the fields. The capability for detecting essential textural details increased by 80.0%, revealing improved cooperation for detecting specific textural variations, which become vital for detecting early-stage crops. Energy reached 63.6% enhancement in the statistical measures, and entropy dropped 34.1%, which indicates more structured and condensed texture representations. The fusion approach yielded substantial enhancements that deliver superior discriminating information for early-stage crop classification through the proposed methodology.

### Classification methods

For the development of classifiers, five deep learning and machine learning methods were employed.

#### Deep neural network

**Fig. 5:**
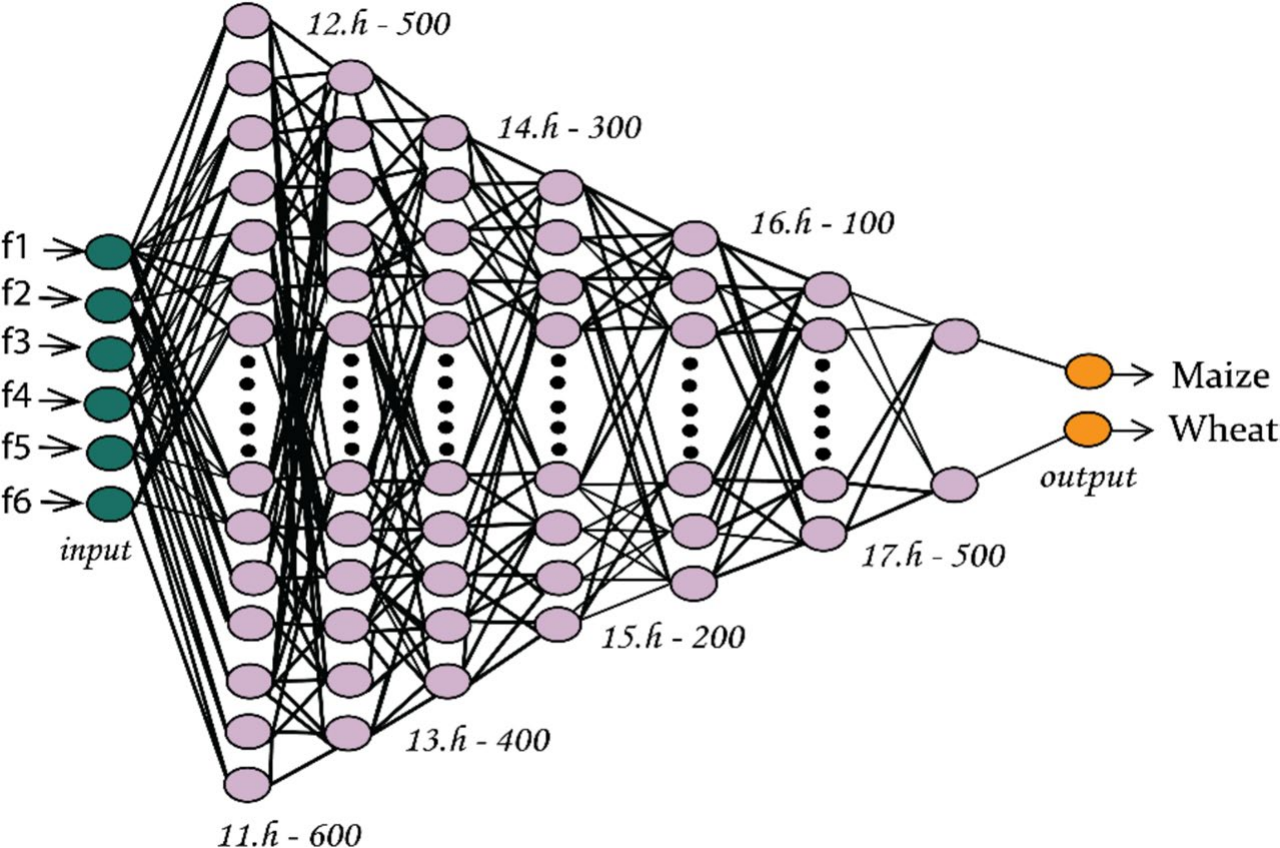
Architecture of DNN

Deep neural network (DNN) belongs among the most effective deep learning algorithms for picture categorization. It can learn complicated patterns and features from the incoming data since it has numerous hidden layers of coupled neurons. Automatic hierarchical representation extraction from visual features is possible with the DNN. The benefit of end-to-end learning, where the model learns to extract pertinent characteristics from the raw data, makes it well-suited for fusion-based techniques (Fig. 5).

#### 1D Convolutional Neural Network

The 1D convolutional neural network (1D Conv) is a variation of the conventional convolutional neural network (CNN) that is specifically made for processing one-dimensional input, such as time series or sequence data. To extract temporal correlations and patterns from the multi-temporal remote sensing data, the 1D Conv can be used. Convolutional processes are used by the 1D Conv to extract features that are important for classifying early-stage crop types.

#### Decision tree

A decision tree is a straightforward yet effective machine-learning technique that creates a decision-making model that resembles a tree. As opposed to the leaf nodes, which represent classification labels, each internal node of the tree represents a choice based on a particular attribute. Using fused features gathered from several sources, a decision tree can be utilized to make decisions in early-stage crop categorization.

#### Support vector machine

An effective machine-learning approach for classification problems is called the support vector machine (SVM). SVM seeks out the best hyperplane in the feature space that maximally divides the classes into distinct groups. SVM can be used to categorize early-stage crops based on fused features received from many sources, such as spectral and textural data.

#### Random forest

Random forest is an ensemble learning technique that blends various decision trees to produce predictions. A randomly chosen portion of the training data and characteristics are used to construct each decision tree in the forest. Random forest can be used to incorporate the fusion features collected from several sources in the context of early-stage crop categorization. It can handle high-dimensional data and record non-linear correlations between features.

#### LightGBM

LightGBM is a gradient-boosting decision tree algorithm which has been specifically developed for large-scale data and categorical features. It makes use of histogram-based methods to achieve less memory usage as well as faster training as compared to the other two. framework based on decision tree algorithms, optimized for speed and efficiency, particularly in handling large datasets and categorical features. It uses histogram-based methods to reduce memory usage and increase training speed.

#### XGBoost

XGBoost is an optimized, reliable gradient-boosting platform developed for handling large-scale structured data and tabular structures. Extremely fast and accurate with the ability to add a regularization step to improve model generalization ability in high-dimensional space, XGBoost is ideal for large and detailed inputs and accuracy. XGBoost integrates regularization to prevent overfitting, making it well-suited for complex, high-dimensional datasets.

#### CatBoost

CatBoost (categorical boosting) is a gradient boosting technique that does not require categorical features to be transformed, which makes CatBoost different. Being a powerful tool for working with a great number of categorical variables and using GPU for computations, CatBoost shows high accuracy and results on the given datasets. Handling categorical features and GPU support, CatBoost provides high accuracy and performance on datasets with numerous categorical variables.

### Evaluation metrics

Confusion matrix is a very popular measure used for the evaluation of classifiers. The structure of the confusion matrix for binary classification is presented in Table 7.

**Table 7:** Confusion matrix for binary classification

	Actual class		
		Positive	Negative
Predicted class	Positive	True positive (TP)	False positive (FP)
	Negative	False negative (FN)	True negative (TN)

The data from the confusion matrix are used for the calculation of some evaluation metrics. In this study, the two classes are recognized, namely wheat and maize. Therefore, the parameters TP, FP, FN, and TN should be interpreted in this context. The TP (the object is predicted positive and is positive) is a wheat crop recognized as a wheat, FP (the object is predicted positive and is negative) is a maize crop recognized as a wheat, FN (the object is predicted negative and is positive) is a wheat crop recognized as a maize, and TN (the object is predicted negative and is negative) is a maize crop recognized as a maize. The evaluation metrics that are used in this study to evaluate the performances of the classifiers are listed below.

#### Accuracy

Accuracy measures the overall correctness of the classification model and is calculated as the ratio of correctly classified samples to the total number of samples, expressed in Eq. 10.

10 $$\text{Accuracy}=\frac{TP+TN}{TP+TN+FP+FN}$$

#### Precision

Precision measures how well a model can identify positive samples among all the samples that it has predicted to be positive, as expressed in Eq. 11.

11 $$\text{Precision}=\frac{TP}{TP+FP}$$

#### Recall

The model’s capacity to recognize every positive sample is measured by recall. It is determined as the ratio of true positives to the total of true positives and false negatives, as expressed in Eq. 12.

12 $$\text{Recall}=\frac{TP}{TP+FN}$$

#### F1 score

The F1 score is a combination of precision and recall calculated as follows:

13 $${F}_{1}=2\bullet \frac{\text{Precision}\bullet \text{Recall}}{\text{Precision}+\text{Recall}}$$

when a classifier has a high value of F1 score, it means that it has a high value of both precision and recall.

## Results

**Fig. 6:**
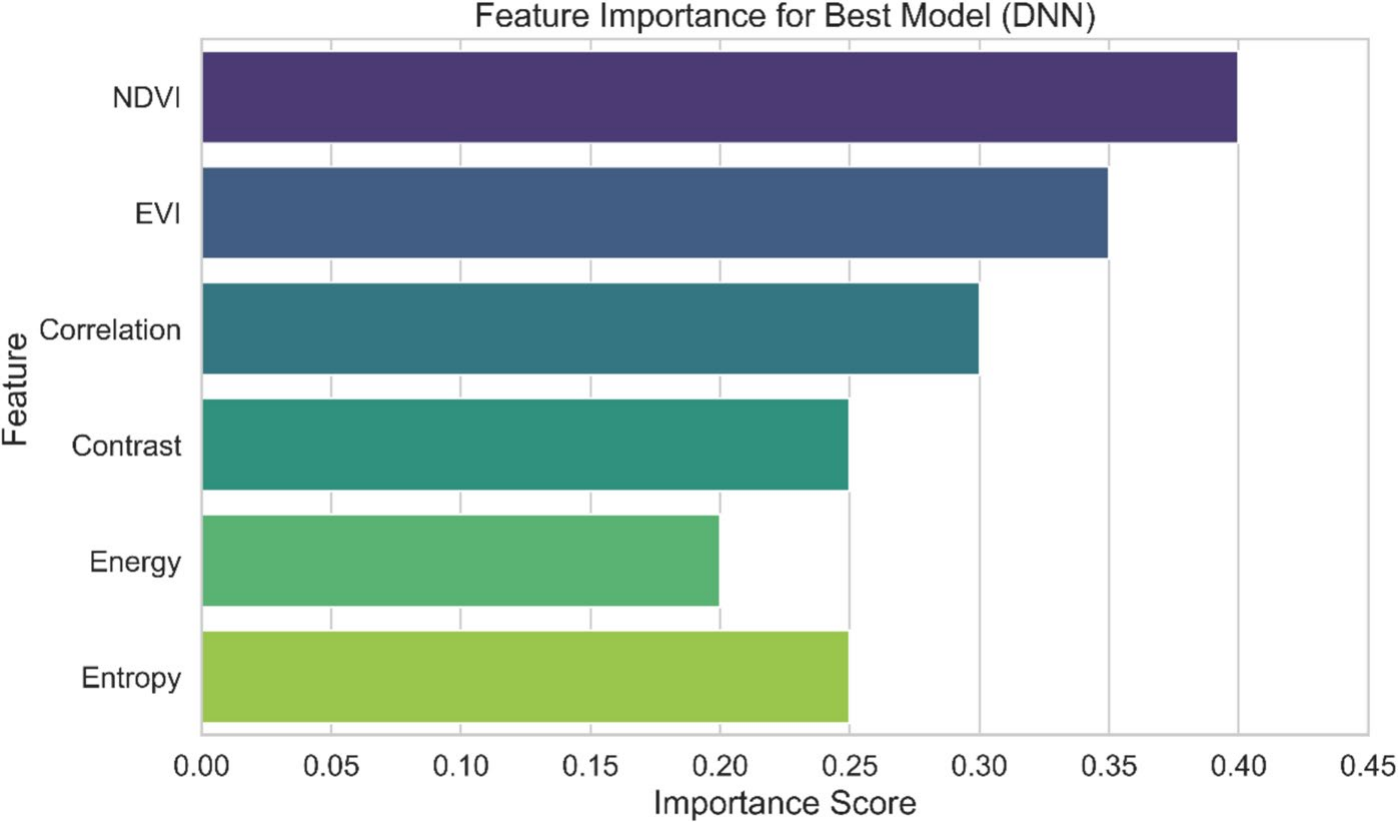
Features importance for best model (DNN)

The proposed fusion approach for early-stage crop classification is evaluated by implementing DNN, 1D-Conv, RF, DT, and SVM methods. A brief detail about the methods is discussed in the “Classification methods” section. The classifiers were trained for the recognition of wheat and maize based on four texture parameters and two spectral indicators described in the “Data preparation” section. The feature importance of the best performance model is presented in Fig. 6. The metrics to evaluate the results are accuracy, precision, recall, and F1 score. The metrics have been discussed in the “Evaluation metrics” section.

Table 8 shows the accuracy achieved by all the models against time series (T1–T5). It shows how the models achieve accuracy over different time series; as the time series increases, the accuracy increases because the characteristics of crops become increasingly clear at the harvest stages.

**Table 8:** Overall accuracy comparison

Models	T1	T2	T3	T4	T5
SVM	0.47	0.64	0.72	0.78	0.82
DT	0.48	0.69	0.73	0.81	0.83
RF	0.48	0.70	0.76	0.84	0.88
1D Conv	0.49	0.75	0.81	0.87	0.91
DNN	0.49	0.76	0.83	0.90	0.93
LightGBM	0.47	0.68	0.74	0.82	0.86
Xgboost	0.48	0.69	0.75	0.83	0.87
CatBoost	0.47	0.67	0.73	0.81	0.85

**Fig. 7:**
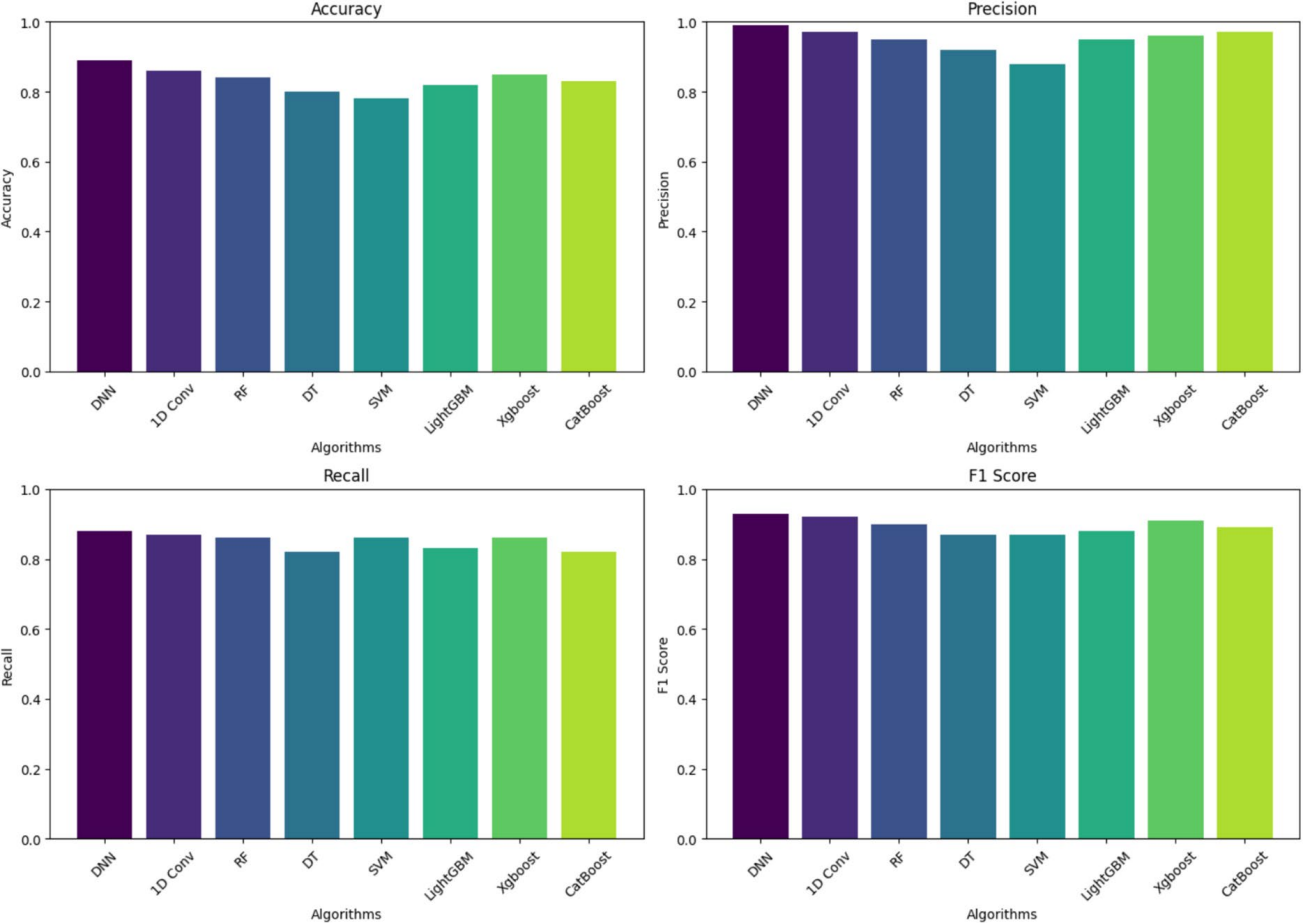
Performance metric comparison at T-4 (accuracy, precision, recall, and F1 score) for various algorithms, clearly comparing how well they perform in classification

Figure 7 shows the last classification maps for time points t1 to t5, which depict the spread of crop types. The figure also has performance metrics (accuracy, precision, recall, and F1 score) for different algorithms, allowing for evaluating the models’ effectiveness. This combined figure improves the understanding of results by showing both the spatial and numerical data together. The longer the time series data, the more accurately crop classification results can be achieved. The accuracy at t1 is low because crops at the very early stages of their growth exhibit very few spectral and textural characteristics to be discriminative. At t4, the results using the fusion-based approach showed exceptional growth in accuracy. T5 accuracy is the one when crops start to exhibit the later growth stages characteristics and become more discriminative, achieving more accuracy. The hyperparameters used during the training of each model are given in Table 9.

**Table 9:** Hyperparameters of each model

Model	Hyperparameters	Description
DNN	Layers, neurons per layer	3, layers with different numbers of neurons were used [128, 256, 128]
	Optimizer, learning rate	It looks like high performance becomes more stable when using the Adam optimizer with a learning rate of 0.0005
	Epochs	Learning these complex features required 120 epochs of training in this case
1D Conv	Filters, kernel size	64 and 128 filters where the kernel size of the filters was set to 3 to capture spatial features
	Optimizer, learning rate	It looks like high performance becomes more stable when using the Adam optimizer with a learning rate of 0.0005
RF	Number of trees, max depth	200 trees with a maximum depth of 10 to reduce high variance and increase stability through time
DT	Criterion, max depth	Gini Criterion, with max depths of 8 used to make consistent predictions
SVM	Kernel, C	RBF kernel with C = 0.5 was used for moderate regularization
LightGBM	Boosting type, leaves, max depth	GBDT boosting with 40 leaves, which gives a depth of 8 for the model to capture more intricate details
Xgboost	Booster, learning rate, max depth	Using a gbtree booster with 0 specifications that include a learning rate of 0.05 and a depth of 6 results in a gradual improvement of the intervals
CatBoost	Depth, learning rate, regularization	It states a depth of 6, a learning rate of 0.03, and an L2 regularization of 3.5 in the second to avoid overfitting and get stability and accuracy from the model

All the models showed exceptional growth in accuracy in the t4 time-series, which is as early as 2 months of the harvest stage of wheat and 5 weeks of maize. Among the methods employed in this study, DNN performed very well in classifying the crop at its early stages. The DNN architecture is shown in Fig. 5.

The DNN used to classify wheat and maize crops at the initial stages consists of seven hidden layers. The more hidden layers do cause overfitting, but fewer layers cause underfitting. Therefore, the RelU activation function was used as it can avoid vanishing gradients and makes training faster. In the output layer, the softmax function was used, which classifies the crops at the end. The data presented in Table 8 shows that among all the classifiers, DNN outperforms them all in the context of early-stage crop classification, achieving an accuracy of 89%, precision of 99%, recall of 88%, and F1 score of 93%. The proposed approach achieved good results in all the models. DNN, 1D Conv, and RF performed better than SVM and DT in terms of accuracy (Table 10).

**Table 10:** t4 accuracy metrics comparison

Algorithm	Accuracy (%)	Precision (%)	Recall (%)	F1 score (%)
DNN	89	99	88	93
1D Conv	86	97	87	92
RF	84	95	86	90
DT	80	92	82	87
SVM	78	88	86	87
LightGBM	82	95	83	88
Xgboost	85	96	86	91
CatBoost	83	97	82	89

**Fig. 8:**
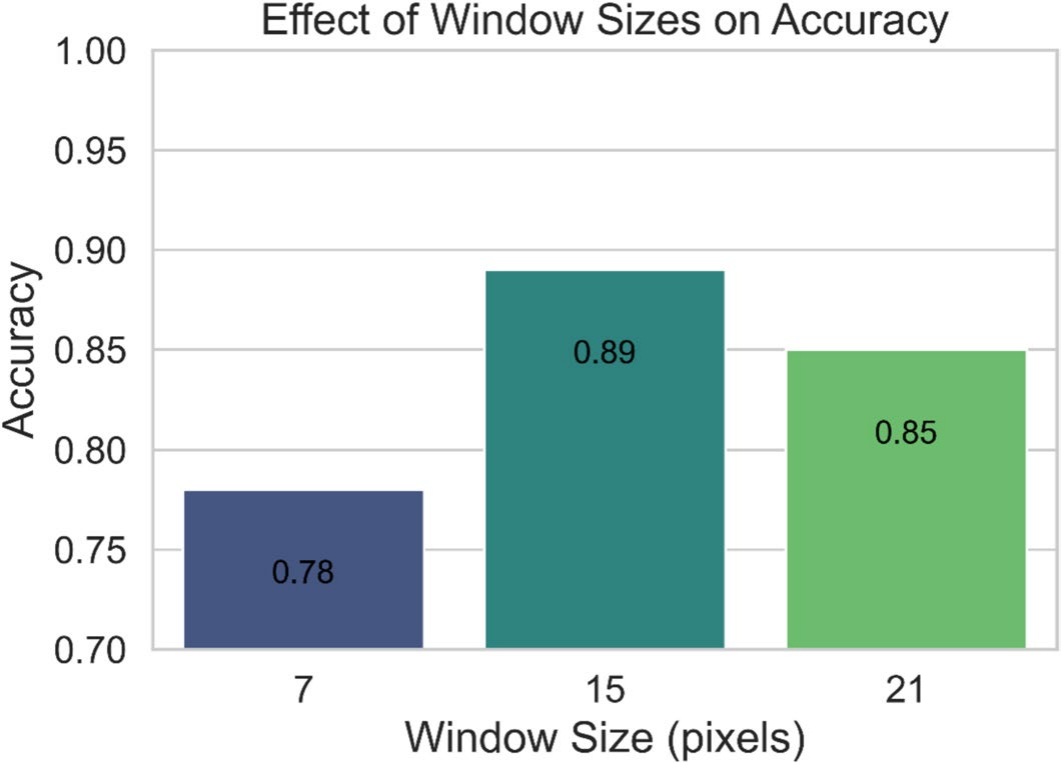
Effect of window sizes on accuracy

To investigate the influence of window size on DNN accuracy, we experimented with three different window sizes: 7, 15, and 21 pixels, as shown below in Fig. 8. The neighborhood of pixels was extracted from each image, which helps to identify the spatial context around every pixel and is important in enhancing classification results. In this process, a square window (neighborhood) is chosen, with the number of pixels across it varying based on the configuration of the particular image (7 × 7, 21 × 21). For a 7-pixel neighborhood, the window size is 7 × 7, with 49 pixels; for a 21-pixel neighborhood, the window size is 21 × 21 and 441 pixels. The spatial features are derived by applying statistical functions on the neighboring pixel values, including mean, standard deviation, and entropy, to the spectral features. This leads to the capture of the local spatial variation, such as the texture as well as the variation in crops. The size of the neighborhood being chosen has a strong effect on the technique’s results. Smaller regions, for instance, the 7-pixel neighborhood, provide more detailed information about an area but are noisy and may fail to detect large-scale spatial structures. More extensive areas, such as the 21-pixel window, have greater contextual information but miss small details to produce a blurry model. The 15-pixel neighborhood (15 × 15) configuration was found to deliver the best balance between spatial detail for image analysis and avoiding over-smoothing for gaining optimal classification accuracy, for example, 89% overall accuracy using DNN. The results derived from our experiments show that window size is vital when it comes to texture depiction and model performance. It was established that the highest accuracy of 89% is reachable with the window size being 15 pixels. A smaller number of pixels (7 pixels) led to lower accuracy (78%) of the system because of less texture information. Whereas large windows (21 pixels) seemed to include excessive noise from the neighboring areas, resulting in lower accuracy (85%). Thus, the size of 15 pixels is chosen as optimal as it provides appropriate texture features while preserving data from noise.

**Fig. 9:**
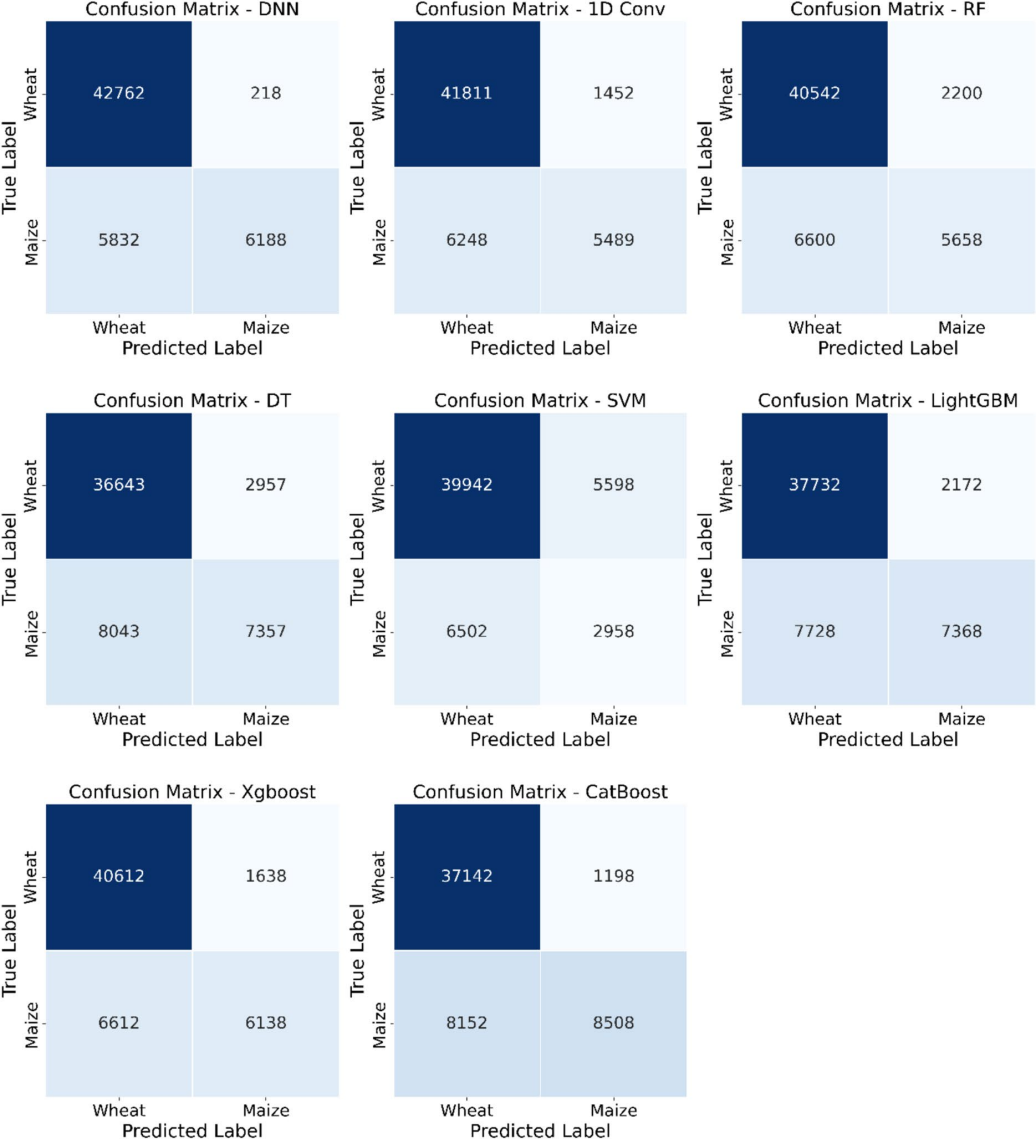
Confusion matrices

In order to improve the performance of the models and give a better and more detailed comparison of the models, a confusion matrix has been included. Figure 9 provides detailed definitions of the correct and incorrect analyses, indicating the strengths and weaknesses of the model.

**Fig. 10:**
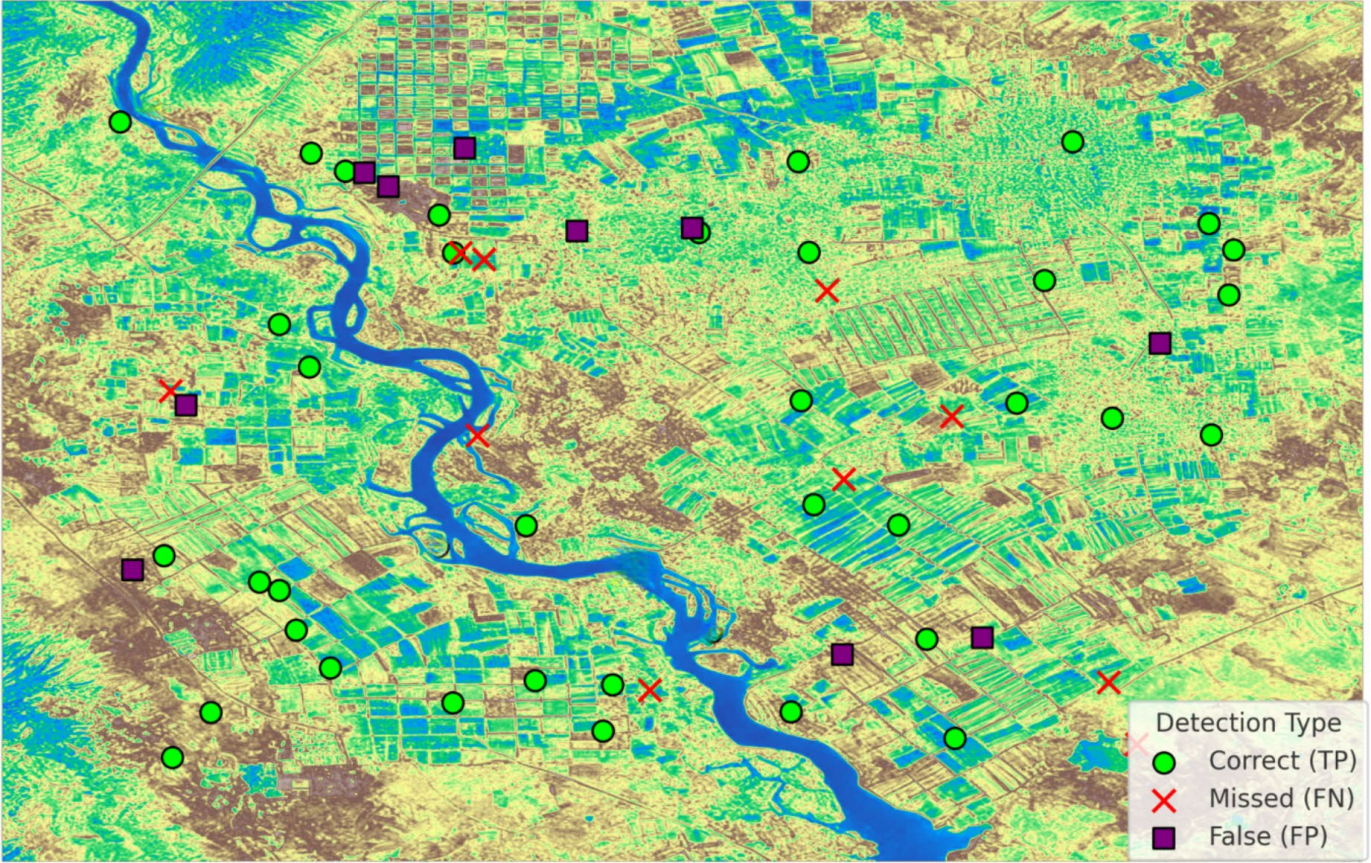
Crop and non-crop object detection

Figure 10 reflects detailed accuracy and error visualization of our crop classification results. It indicates that the correct identification of crop fields through accurate classification methods produces green circular markers.

The visualization shows actual crop fields that the classifier failed to detect through red “X” markers, along with correct identification of crop fields marked by green circles and incorrect classifications shown by purple squares.

The false detections, which falsely classified non-crop areas as crop fields, appear as purple squares within the visualization.

## Discussion

Although some studies indicate better accuracy results for crop and land classification, our proposed approach has one distinctive feature which is very important in the context of monitoring crops during the early stages of growth. Our dual fusion method, which integrates both Gram-Schmidt image fusion and multi-patch GLCM feature fusion, is aimed at improving primary crop classification and detecting features when there is little difference both spatially and spectrally. The Gram-Schmidt fusion method enhances spatial resolution while preserving the multispectral bands’ spectral fidelity, allowing for the retention of high-frequency textural detail and low-frequency spectral information. This method is useful for differentiating crops by stages since most classification models fail because of high spectral similarity among many crops at juvenile stages. The spatial integrity of the fused images assures radiometric integrity so that vegetation indices like NDVI and EVI are still responsive to small changes in crop canopy structure, enabling more accurate discrimination of different crop types at the early growth stages (Wei et al., 2022a, 2022b; Yi et al., 2020).

The addition of multi-patch GLCM into the texture feature extraction process introduces a solid statistical structure. This structure is adept at capturing the nuances of texture variations seen from various spatial angles, namely 0°, 45°, 90°, and 135°. In stark contrast to the traditional single-scale GLCM techniques, the multi-patch method steps up the game by accurately calculating texture descriptors across neighborhoods of differing sizes. This methodical calculation leads to a more refined adjustment to the diverse structures found in crops. To understand the complex patterns within crop fields, characteristics like contrast, correlation, energy, and entropy were pulled out; notably, the contrast levels saw an 80% boost after merging, showing better texture distinction. By combining spectral and textural elements through the Feature Weighting Method, or FWM, the classification task gets fine-tuning. This approach adjusts the importance of each feature by using standard distance measures, making sure the right features stand out (Rahmati et al., 2022).

The examinations of various window size dimensions in GLCM-based texture extraction found that a 15 × 15-pixel window produced the best accuracy levels (89%), exceeding both smaller (7 × 7) and larger (21 × 21) configurations. Window size directly influenced the amount of spectral noise present, together with the ability to preserve crop structure features in the images. The analysis highlights the need to perfect spatial context in texture feature extraction because this helps achieve higher classification precision by reducing spectral similarity and background-related classification errors (Cao et al., 2021; Zhong et al., 2019).

While the proposed fusion approach shows good performance, it faces difficulties when used in different farming areas. The researchers worked in District Vehari, Pakistan, because specific soil and climate factors, along with plant development patterns, are unique to its environment. The project can enhance its results by researching transfer learning models from different farming environments. Hyperspectral imaging and synthetic aperture radar data addition would enhance classification results, especially during times when optical sensors are affected by atmospheric changes, as reported by Demarez et al. (2019). Future research needs to study different ways the method works in locations where farms grow various crops and soil types.

Using the Gram-Schmidt fusion method with multi-patch GLCM features and deep learning creates an effective way to detect crops at an early stage. The new method effectively identifies crop types by combining spectral and textural data, which enables real-time decisions and builds the base for automatic crop monitoring tools.

The results obtained based on the proposed approach using DNN and 1D Conv were superior to the other techniques because of the capability of the approaches in modeling higher-order relationships of fused spectral-textural features (Wang et al., 2022; Zhang et al., 2021). A complex DNN architecture using four hidden layers was able to learn about feature hierarchies by capturing both spectral features, in terms of long-range spatial correlation, and local textural features within the dataset. Compared to most traditional machine models, such as random forest and decision tree, deep learning models automatically learn the feature hierarchy and, therefore, a higher recognition accuracy of the crop at early stages (Sun et al., 2019; Xia et al., 2022). The results also showed that as time-series data covered more time (T3-T5), classification accuracy increased, and the capability of feature learning based on multi-temporal data allows for better differentiation of the phenological crop growth stages (Yi et al., 2022).

In this research, the evaluation of the suggested strategy using DNN and 1D Conv showed promising accuracy results. Better categorization performance was made possible by the combination of spectral and textural data, which gave a complete picture of the early-stage crops. It is crucial to understand the limits of this study, though. The findings may not be immediately transferable to other places with varied crop types and environmental circumstances because the study was restricted to District Vehari in Pakistan. This strategy has a tremendous amount of potential for enhancing crop management techniques, streamlining resource distribution, and promoting precision agriculture.

## Conclusion and future work

The current study concentrated on a particular study region and crop varieties. To evaluate the generalizability of the suggested approach across various agricultural contexts, transfer learning methods need to be explored. The next study may involve expanding the research to encompass a larger range of crop kinds and various geographic locations. To further improve the discriminative power and accuracy of the crop classification models, future research might investigate the incorporation of multi-modal data, such as hyperspectral images or UAV data, in addition to spectral and textural variables.

This strategy has a tremendous amount of potential for enhancing crop management techniques, streamlining resource distribution, and promoting precision agriculture.

Furthermore, future work can be extended to the crop harvest stage classification to this range to maximize its usefulness to the agricultural industry. To achieve this, we will have to employ state-of-the-art machine learning methodologies and may work with additional data modalities, such as climatic data and soil health indices. We are optimistic that such improvements would be immensely beneficial to the agricultural industry by providing farmers with the desirable information to help them maximize production and effects on yields.

## Data Availability

Data are available upon request from the first corresponding author, Dr. Ali Zakir (zakirali@cuivehari.edu.pk).
